# A Mobile Phone–Based Self-Monitoring Tool for Perioperative Gastric Cancer Patients With Incentive Spirometer: Randomized Controlled Trial

**DOI:** 10.2196/12204

**Published:** 2019-02-19

**Authors:** Ji Yeong Soh, Se Uk Lee, Inpyo Lee, Ki Sang Yoon, Changho Song, Nam Hun Kim, Tae Sung Sohn, Jae Moon Bae, Dong Kyung Chang, Won Chul Cha

**Affiliations:** 1 Department of Digital Health Samsung Advanced Institute for Health Sciences & Technology Sungkyunkwan University Gangnam-gu Republic of Korea; 2 Department of Emergency Medicine Seoul National University Hospital Seoul Republic of Korea; 3 Product Development Division BreaThings Co, Ltd Seoul Republic of Korea; 4 R&D Division BreaThings Co, Ltd Seoul Republic of Korea; 5 Camera R&D Group, Samsung Electronics Seoul Republic of Korea; 6 Department of Surgery Samsung Medical Center Sungkyunkwan University School of Medicine Seoul Republic of Korea; 7 Department of Gastroenterology Samsung Medical Center Sungkyunkwan University School of Medicine Seoul Republic of Korea; 8 Health Information and Strategy Center Samsung Medical Center Seoul Republic of Korea; 9 Department of Digital Health Samsung Advanced Institute for Health Sciences & Technology Sungkyunkwan University Seoul Republic of Korea; 10 Department of Emergency Medicine Samsung Medical Center Sungkyunkwan University School of Medicine Seoul Republic of Korea

**Keywords:** incentive spirometer, mobile health, postoperative care, gastric cancer, motivation

## Abstract

**Background:**

An incentive spirometer (IS) is a medical device used to help patients improve the functioning of their lungs. It is provided to patients who have had any surgery that might jeopardize respiratory function. An incentive spirometer plays a key role in the prevention of postoperative complications, and the appropriate use of an IS is especially well known for the prevention of respiratory complications. However, IS utilization depends on the patient’s engagement, and information and communication technology (ICT) can help in this area.

**Objective:**

This study aimed to determine the effect of mobile ICT on the usage of an IS (Go-breath) app by postoperative patients after general anesthesia.

**Methods:**

For this study, we recruited patients from April to May 2018, who used the Go-breath app at a single tertiary hospital in South Korea. The patients were randomly classified into either a test or control group. The main function of the Go-breath app was to allow for self-reporting and frequency monitoring of IS use, deep breathing, and active coughing in real time. The Go-breath app was identical for both the test and control groups, except for the presence of the alarm function. The test group heard an alarm every 60 min from 9 am to 9 pm for 2 days. For the test group alone, a dashboard was established in the nurse’s station through which a nurse could rapidly assess the performance of multiple patients. To evaluate the number of performances per group, we constructed an incentive spirometer index (ISI).

**Results:**

A total of 44 patients were recruited, and 42 of them completed the study protocol. ISI in the test group was 20.2 points higher than that in the control group (113.5 points in the test group and 93.2 points in the control group, *P*=.22). The system usability scale generally showed almost the same score in the 2 groups (79.3 points in the test group and 79.4 points in the control group, *P*=.94). We observed that the performance rates of IS count, active coughing, and deep breathing were also higher in the test group but with no statistically significant difference between the groups. For the usefulness “yes or no” question, over 90% (38/42) of patients answered “yes” and wanted more functional options and information.

**Conclusions:**

The use of the Go-breath app resulted in considerable differences between the test group and control group but with no statistically significant differences.

**Trial Registration:**

ClinicalTrials.gov NCT03569332; https://clinicaltrials.gov/ct2/show/NCT03569332 (Archived by WebCite at http://www.webcitation.org/74ihKmQIX).

## Introduction

### Background

Vigorous and multidisciplinary efforts are required to prevent postoperative complications following prolonged general anesthesia [1], and an incentive spirometer (IS) plays a key role, especially for patients with limited mobility [2]. An incentive spirometer is a medical device used to help patients improve the functioning of their lungs. It is provided to patients who have had any surgery that might jeopardize respiratory function. The IS is used for the prevention of atelectasis, hypoxemia, pneumonia, respiratory dysfunction, and pleural effusion [3,4]. However, IS utilization has remained the same since its initial development in the 1960s, and the usage protocol has not been standardized [5-8].

Although many mobile phone apps have been developed for self-management in various clinical settings, only a few cases showing their use in postoperative care were reported [9], even though subjective activity by patients can influence their clinical hospital outcomes. Therefore, with help from mobile information and communication technology (ICT), patients can be more engaged in behaviors that can lead to better clinical results [3,10,11].

A mobile ICT for hospitalized postoperative patients can improve clinical outcomes by several pathways: it can encourage patients by informing them of important performance indices such as exercise duration [12-14], and it can connect the patient and provider with a real-time dashboard that can support prompt provider reactions. In a previous study, a postoperative group of patients using mobile ICT recovered significantly better than a control group [9].

### Objective

Therefore, the goal of this study was to determine the effect of mobile ICT on the performance of postoperative patients using an IS (Go-breath) app after general anesthesia.

## Methods

### Study Design

This was a single-center, randomized controlled trial (RCT) assessing the effectiveness of an IS self-reporting app (Go-breath) to improve IS performance and effectiveness. This trial was registered with the US National Institutes of Health Clinical Trials Registry (NCT03569332).

### Study Setting

This study was conducted at an academic tertiary center located in Seoul, Korea. The hospital’s expertise is cancer care. It hosts approximately 2000 inpatient beds and registers approximately 8500 outpatients per day.

Gastric cancer is one of the major diseases treated in the institution. The hospital’s Critical Pathway defines the routine care process for gastric cancer surgery as follows: The patient is admitted the evening before the surgery and discharged approximately 9 to 10 days after surgery—the discharge period of laparotomy is postoperative day (POD) 8 and for laparoscopy is POD 7—without complications; the patient receives instructions on the routine use of an IS before the surgery, and its use is initiated the morning after the surgery (POD 1).

### Study Participants

Participants were enrolled from April to May 2018. We recruited patients from the surgical ward immediately after the admission process. Our eligibility criteria stipulated that patients were older than 18 years and planned to undergo robotic or laparoscopic surgery or a laparotomy. The enrollment was performed the day before the surgery, and the intervention was initiated the day after the surgery (POD 1). Following recruitment, participants were provided written informed consent and randomized using a Web-based random number generator and sealed enveloped in a 1:1 ratio. If the patient was in the intensive care unit (ICU) or in an emergency condition requiring nonelective surgery, he or she was excluded from the study. The patients were dropped from the study if the postoperative state of the patients was not difficult to predict in advance and if their postsurgical condition (and before the initiation of intervention) was consistent with the exclusion criteria such as ICU admission. Dropout was only permitted on the day of surgery (POD 0).

### Study Protocol

Patients were allocated to the test and control groups after giving their consent for the study. Patients received a tablet with the Go-breath app installed, and they received information about the app and use of the IS for approximately 30 min. The IS awareness session was carried out using either video clips (Multimedia Appendix 1) or nurses as part of the routine clinical process.

Generally, the nurses were encouraged to check the performance of the IS on the patients over 10 times every hour after the surgery, and some ward nurses checked the IS performance rate more than 10 times per hour from 9 am to 9 pm.

The Go-breath app was identical for both the test and control groups except for the alarm function. The test group heard an alarm every 60 min from 9 am to 9 pm during POD1-POD2 (a total of 24 hours). For the test group only, a dashboard was established in the nurse’s station through which a nurse could rapidly assess the performance of multiple patients. On the other hand, the control group was not provided an alarm function or nurse dashboard identification. The control group only entered the number of IS performances using the app.

**Figure 1:**
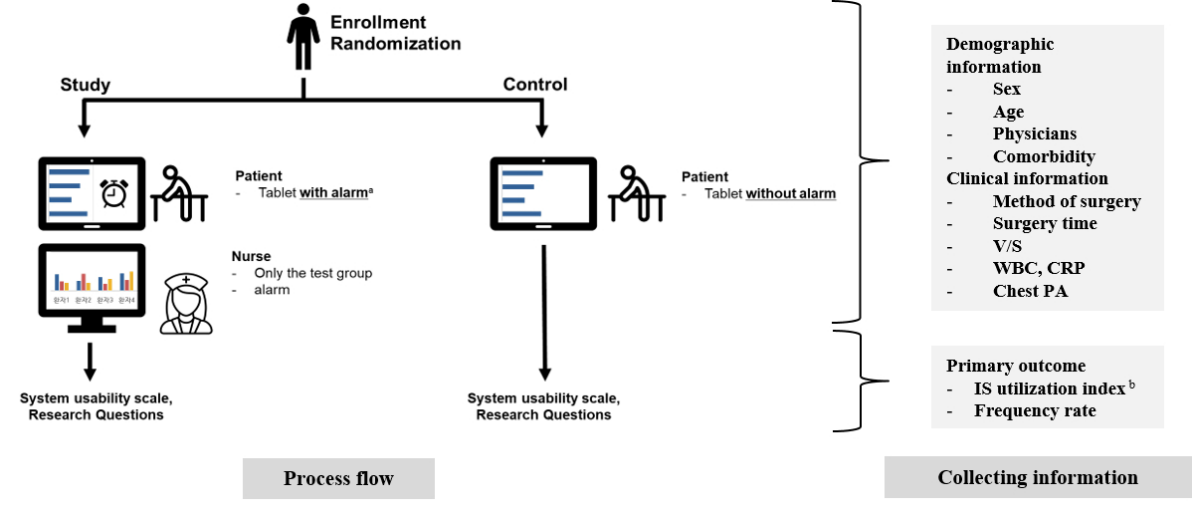
Study protocol. Superscripted "a" indicates alarm every 50 min for insufficient during 9am-9pm (alarm off during 9pm-9am); "b" indicates calculation of performance score applies only to incentive spirometer (full score 240, 10 out of 10 points). V/S: blood pressure, fever, body temperature, respiration rate; CRP: C-reactive protein; IS: incentive spirometer; PA: posterior to anterior; WBC: white blood cell.

The measurement of IS use was unlimited, but the record of the Go-breath app and dashboard would show up to a maximum of 10 times per hour. We named this the incentive spirometer index (ISI), and a description of this index is provided in the major outcomes section. Termination of the study occurred in the morning of POD 3. Study assistants visited each patient, collected devices, conducted a short survey about usability among the patients and interviewed the patients for subjective opinions. Even though the study intervention lasted for only 2 days, the patients’ outcomes were tracked until the day of discharge. An overview of the study protocol is provided in Figure 1.

### Device and System

#### Hardware and System Architecture

**Figure 2:**
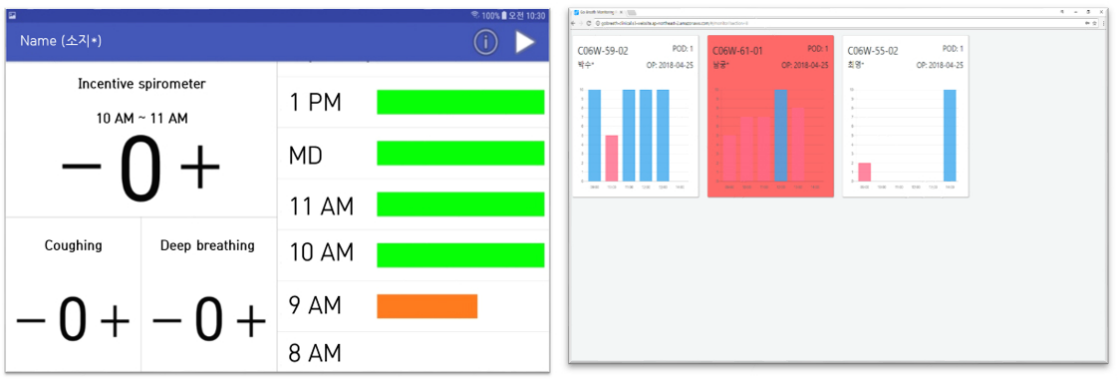
Screenshot of the Go-breath app (left) and dashboard (right). The original version was in Korean, but it was modified to display English.

The system was constructed based on the opinions of health providers and comprised a mobile phone app for patients and a server for medical staff. Patients could self-input their performance of IS, active coughing, and deep breathing every time. This information was transmitted to a nurse dashboard for a real-time check. All patients were given the same hardware (Galaxy tablet A 8.0, Samsung, purchased 2018) running Android 8.0, and the mobile Go-breath app was tested on Android 8.0 utilizing an Amazon Web service server (Figure 2).

#### Go-breath App

**Figure 3:**
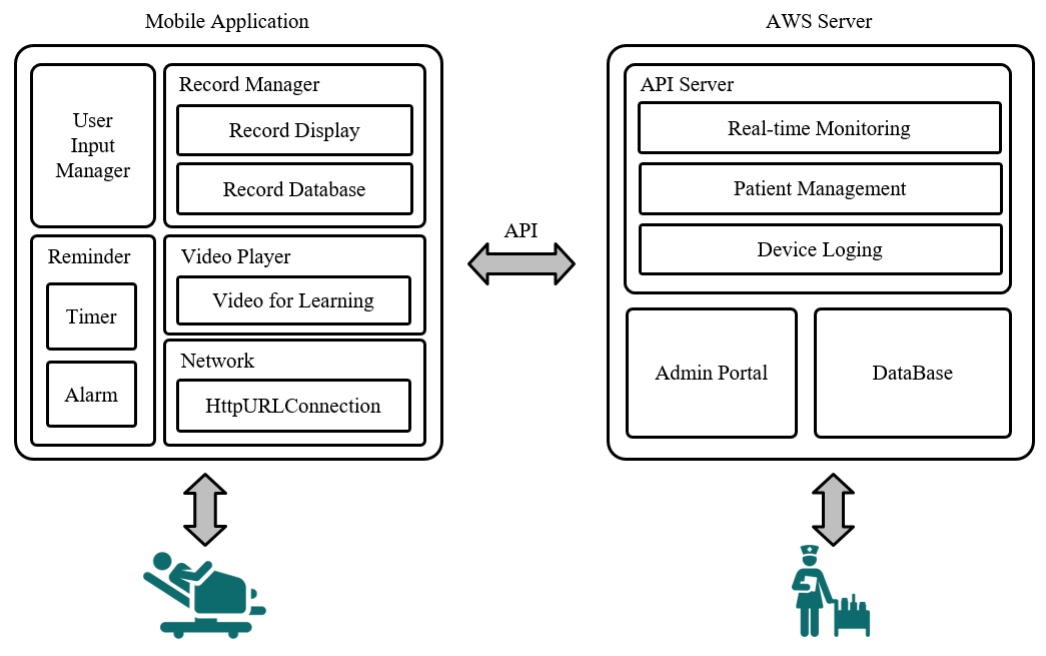
System architecture. API: application programming interface; AWS: Amazon Web Service.

The main function of the Go-breath app was to allow for self-reporting and frequency monitoring of IS, deep breathing, and active coughing in real-time. In principle, patients were instructed to use the IS 10 times every hour and simultaneously report it to the app. The optimal number of times to use the IS was derived from the literature [2,6]. Patients could also check on their current state and edit previous indices they had input (Figure 3).

The Go-breath app was set to alarm when the number of IS uses did not reach 10 in an hour. The app was set to ring 10 min before the end of each hour to give the patients time to complete the expected number of hourly uses. When the patients fulfilled their hourly IS use, the alarm would silence automatically. However, patients could silence the alarm. When the mute icon was pressed, the alarm would be deactivated for 2 hours and would reactivate by default. This functionality was provided because some of the patients could be away from their bed for various reasons, and the alarm could disturb other patients. The system remained frozen during the study period. We had preserved the anonymity of the patients by an unidentified number.

#### Go-breath Dashboard

Only information from the test group was displayed on the Go-breath dashboard. The dashboard showed IS performance that did not include other measures, such as deep breathing and active coughing. Each patient was displayed as a card with his or her location and POD information. For each card, the patient’s ISI during the most recent 6 hours was displayed as a bar graph. If the most recent hourly ISI was not optimal (below 10), the card turned red. The timing and alarm rules were identical to that of the Go-breath app (Figure 3).

### Major Outcomes

#### Primary Outcome

We developed the ISI, which ranged from 0 to 120 per day. The maximum score was 240 for a 2-day observation period. We developed an outcome index using clinical experts and supporting literature [2,6-8]. The ISI was defined as 10 points per each hour, the frequency of IS use was 10 times or more for that hour. If the frequency was below 10 for that hour, the score was rated as 0. ISI was only measured from 9:00 am to 9:00 pm at PODs 1 and 2. We compared the ISI of the test group and control group.

#### Secondary Outcome

The secondary outcome was the System Usability Scale (SUS). At the end of the study, patients completed an offline questionnaire to evaluate the system using the SUS, which is widely used in health information technology research [15-20]. The questionnaire measures the usability of hardware and software products, and it comprises only 10 items that respondents score on a 5-point Likert scale. Answers were converted to a final 0- to 100-point score, and the higher the score, the more usable the product [17,19,21].

#### Other Outcomes

In addition to the major outcomes, we measured IS use frequency, active coughing frequency, deep breathing, and clinical results (as length of stay). Clinical data, such as surgery, laboratory data including white blood cell, C-reactive protein, vital signs (fever), and chest posterior to anterior, and length of stay were also collected.

#### Interviews

After the study, a brief face-to-face interview with every patient was performed by a research assistant. Patients answered whether the system was helpful or not and rated the score. Patients’ subjective opinions of the system were also gathered during the process.

### Data Collection

We collected the patients’ baseline demographic information along with variables associated with surgery including surgeon, type of surgery, and duration of the operation. In the trial, all participants were asked to record log when they started and finished Go-breath app every day. Missing entries were interpreted as a missed value.

### Sample Size Calculation

We calculated the sample size of this study with the G-power 3.1 program (Statistical power analyses using G*Power 3.1 [22]). Several assumptions were made to set the sample population. The power was set to 0.90, significance level was set to .05, and effect size was 1.00. The minimum number of samples for the Wilcoxon rank-sum test was 19 in each group. We assumed that the ISI would be different by 60% (12/20; test group) versus 40% (8/20; control group), with SDs for each group. This assumption was made based on expert opinions because there was no evidence that described IS frequency. Under these conditions, the sample size was 19 patients, and finally, 22 patients were selected for each group considering a dropout rate of 10%.

## Results

### Study Participants

We recruited 44 patients from April to May 2018, with 22 patients allocated to the test group and 22 patients allocated to the control group from patients who used the Go-breath app at a single tertiary hospital in South Korea. A total of 2 patients were excluded because of clinical conditions. The overall average age was 55.9 (SD 12.9) years. The proportion of female patients was 34.1%. All patients had gastric cancer, and the overall average surgery time was 169 (SD 46.2) min. The demographic information of both groups is shown in Table 1.

### Major Outcomes

The primary ISI outcome and the secondary SUS outcome are shown in Table 2, and no statistically significant differences were noted in these outcomes between the test and control groups. SUS of both groups showed good to excellent grade [21].

For the performance measures, both the IS rate count and deep breathing showed a higher performance without a statistical significance. However, active coughing showed a significantly higher performance in the test group compared with the control group. For the clinical results, including inflammatory markers and pulmonary complications, there were no statistically significant differences between the test and control groups (Table 3).

### Interviews

For the usefulness questionnaire, 90.0% of the test group and 90.9% of the control group answered yes. There were 3 domains of opinions: function addition, function alteration, and etc. The 2 most wanted functions were exercise tracking and peer patients’ information, and the most frequent complaint was about Wi-Fi connection issues (Table 4).

**Table 1:** Demographic characteristics of patients in the test and control groups.

Category		Test group (N=22)	Control group (N=22)
**Sex, n (%)**			
	Female	6 (27)	9 (40)
	Male	16 (72)	13 (59)
Dropout, n (%)		2 (9)	0 (0)
**Age (years), n (%)**			
	30-40	1 (4)	4 (18)
	50-60	12 (54)	11 (50)
	70-80	8 (36)	7 (31)
	Over 80	1 (4)	0 (0)
**Method of surgery, n (%)**			
	Laparotomy	10 (45)	8 (36)
	Laparoscopy, robotics	12 (54)	14 (63)
**Physicians, n (%)**			
	Dr K	9 (40)	7 (31)
	Dr B	3 (13)	3 (13)
	Dr S	5 (22)	5 (22)
	Dr L	5 (22)	7 (31)
**Comorbidity, n (%)**			
	Tuberculosis	0 (0)	1 (4)
	Diabetes mellitus	2 (8)	2 (9)
	Hypertension	4 (20)	8 (36)
	Others	2 (10)	2 (9)
Time of surgery^a^ (min), mean (SD)		168 (54)	170 (36)

^a^Time of surgery was defined as the time from anesthesia to extubation time.

**Table 2:** Major outcomes.

Index	Test group (N=20), mean (SD)	Control group (N=22), mean (SD)	*P* value
ISI^a^	113.5 (0.8)	93.2 (71.2)	.22
SUS^b^	79.25 (20.59)	79.43 (18.83)	.94

^a^ISI (incentive spirometer utilization index) is a score of 10 points per hour if over 10 times from 9 am to 9 pm for 2 days, with a total maximum score of 240.

^b^SUS: System Usability Scale.

**Table 3:** Other outcomes.

Category		Test group (N=20)	Control group (N=22)	*P* value
**Performance rate, mean (SD)**				
	Incentive spirometer, count	139.5 (61.2)	119.0 (84.5)	.27
	Active coughing	79.0 (52.78)	44.1 (43.62)	.04
	Deep breathing	107.8 (66.8)	94.8 (88.33)	.49
**Laboratory results, mean (SD)**				
	WBC^a^, k/mL	6.2 (2.8)	5.9 (1.8)	.93
	CRP^b^, mg/dL	8.1 (4.0)	8.2 (2.9)	.07
Length of stay, mean (SD)		10.8 (1.6)	10.40 (1.00)	.57
**Chest PA^c^, n (%)**				
	Atelectasis	2 (10)	3 (15)	.64
Fever^d^, n (%)		6 (30)	10 (45)	.79

^a^WBC: white blood cell.

^b^CRP: C-reactive protein.

^c^PA: posterior to anterior.

^d^Fever was defined as 37.5ºC (99.5ºF), and it means the number of people who have been checked for fever at least once.

**Table 4:** Components of the qualitative interview.

Part	Contents	Responses (n)
Function addition (N=12)	“I would like to have the ability to check the number of steps when walking after surgery.”	3
	“I would like a report function on the results of my performance compared with other patients and checking the number of balls up.”	3
	“I wish I could hang it on a portable pole and small size device.”	2
	“I would like to be provided with the training methods for coughing and deep breathing in addition.”	1
	“I wish that the software has more fun-factors.”	1
	“I wish my IS use data gets entered automatically.”	1
	“I would like to have a medication schedule on the device.”	1
Alert function	“I wish there was a notice function when the goal is achieved.”	2
	“I would like to be able to customize the alarm settings myself.”	1
	“I was very worried that the alarm would be annoying to other patients.”	1
Other components	“I did not want to use it because of Wi-Fi connection issues.”	3
	“The device and solution were somewhat difficult for the elderly.”	3
	“I expected the nurses to inform me when my performance was not good, only to be disappointed when they did not.”	2

## Discussion

### Principal Findings

The purpose of this study was to assess the effectiveness of a mobile phone app on improving the performance of the IS by postoperative patients. The major intervention of this study was to provide an interactive feedback system for patients to improve their breathing exercise behaviors. To our knowledge, this is the first RCT that involves the IS and a mobile phone app even though multiple research studies had previously shown their feasibility [9,23]. We enrolled 42 patients, split between a test and control group, and compared their ISI, which showed an approximate 20% difference between the 2 groups (113.5, SD 50.8 vs 93.2, SD 71.2), although the difference was not statistically significant.

### Strength and Limitations

The negative outcome could have been influenced by several factors. First, the SDs were very high in both groups. This means the study population was heterogeneous with respect to IS use, which was not considered when designing the clinical trial. To the authors’ knowledge, there was no literature with specific measures of the IS. The SD was higher than other in-hospital indices, which was hard to foresee before the trial. In general, the evidence on the effectiveness of this app is limited and needs further investigation before implementation in mobile health care. Recent studies have shown a substantial variation in the effectiveness of mobile health apps. Previous studies have not reported a significant difference in clinical outcomes in mobile health research because of the difficulties in RCT settings, such as small sample sizes and low levels of research design. However, an effective clinical assessment of mobile health is hard to achieve, and there have been various attempts to determine health engagement and behavioral factors [24,25].

The second possible reason was that the feedback mechanism could have influenced the control group more than intended. Patients behave differently because of several factors, and many patients come from a nonmedical background. However, studies involving the behaviors of hospitalized patients still need more exploration. For example, the Diabetes Prevention Program was introduced to Mobile App America by insurance companies. Mobile App America is a company with proven effective mobile phone app development, and it focuses on participant engagement and app value [26-29]. Individuals involved in technological development, engineering, and theory tend to focus on the technology itself [30]. However, health care user engagement in a postoperation setting includes unpredictable factors such as caregiving, word setting, Wi-Fi connections, and nurse feedback.

The third possible reason could be the effectiveness of the alarms. Many patients were sharing rooms with other patients and were very concerned about the sound of the alarm bothering others. To make this kind of trial more successful, patient feedback should be carefully considered.

Nevertheless, the effect of the mobile phone app was demonstrated along with the details of the ISI and performance rate. All results were based on the difference between the test and control groups. Although the effectiveness of the app was not proven, the possible utility of a mobile phone app in the postoperation setting was shown. In addition, this was the first trial to collect real-time evidence of IS device use over a long duration. Although we could not find a standard for how patients should perform the IS, this research is significant by itself.

The negative outcome of this study is because of the diversity of the patients’ behavior than the effectiveness of the intervention. Both the test and control groups showed a wide SD, which diminished the statistical power. This could have been prevented by appropriate sample size calculations, but to the authors’ knowledge, no other study had revealed such a wide difference in hospitalized patients’ behavior with postoperative conditions.

The reason we had negative outcomes in the SUS could vary. Though we wanted to make an interactive system and evaluate its utility, a majority of the device’s functions were very similar in both groups. From the users’ aspect, reporting on the system could have been the main task. The alarm function was a major intervention for recognition, but it could be a minor task for patients.

This study did not detect a statistically significant difference with Go-breath app usage between the test group and the control group. However, considerable differences were still observed between the 2 groups, and such differences may demonstrate the possible effects of ICT on patient IS engagement. The surgical procedures, comorbidities (Multimedia Appendix 2), and length of anesthesia could have also influenced the outcome, which must have been minimized by randomization.

This study had some limitations. First, we could not measure the frequency of the use of the Go-breath dashboard because of the difficulty of implementing it without substantially disturbing the clinical routines of the acting nurses. Second, because our system relied on the input from a number of patients (nonblinded), the results could have been biased by the patients’ subjective cognition, willingness, or adherence to digital devices. The existence of family members could have also influenced the input. However, even with these potential biases, inputting the data itself could have positively influenced the use of IS, as with other mobile solutions [22]. The third limitation is related to the second one; the control group could have been substantially influenced by the device when inputting IS use. Even without its alarm function, individuals in the test group could see their current IS usage status, which could have encouraged them to use it more frequently, aggravating the Hawthorne effect. Finally, there was no confirmed function for the performance rate. Although we made it possible to modify the number of previous performances, it did not guarantee the accuracy of the number of IS uses. To improve this, a sensor that automatically measures the number of IS uses could provide more accurate data.

### Conclusions

The use of the Go-breath app resulted in considerable differences between the test group and control group but with no statistically significant differences.

## Appendix

Multimedia Appendix 1 Video clips for incentive spirometer after surgery.

Multimedia Appendix 2 Co-morbidities in patients undergoing open surgery and robotic or laparoscopic procedures.

Multimedia Appendix 3 CONSORT‐EHEALTH checklist (V 1.6.1).

## Abbreviations

ICT: information and communication technology
ICU: intensive care unit
IS: incentive spirometer
ISI: incentive spirometer index
NRF: National Research Foundation of Korea
POD: postoperative day
RCT: randomized controlled trial
SUS: System Usability Scale

## References

[1] García-Miguel FJ, Serrano-Aguilar PG, López-Bastida J (2003). Preoperative assessment. Lancet.

[2] Restrepo RD, Wettstein R, Wittnebel L, Tracy M (2011). Incentive spirometry: 2011. Respir Care.

[3] Sebio R, Yáñez-Brage MI, Giménez-Moolhuyzen E, Valenza MC, Reychler G, Cahalin LP (2016). Impact of a pre-operative pulmonary rehabilitation program on functional performance in patients undergoing video-assisted thoracic surgery for lung cancer. Arch Bronconeumol.

[4] Kumar AS, Alaparthi GK, Augustine AJ, Pazhyaottayil ZC, Ramakrishna A, Krishnakumar SK (2016). Comparison of flow and volume incentive spirometry on pulmonary function and exercise tolerance in open abdominal surgery: a randomized clinical trial. J Clin Diagn Res.

[5] Malik PR, Fahim C, Vernon J, Thomas P, Schieman C, Finley CJ, Agzarian J, Shargall Y, Farrokhyar F, Hanna WC (2018). Incentive spirometry after lung resection: a randomized controlled trial. Ann Thorac Surg.

[6] Hall JC, Tarala RA, Tapper J, Hall JL (1996). Prevention of respiratory complications after abdominal surgery: a randomised clinical trial. Br Med J.

[7] Westwood K, Griffin M, Roberts K, Williams M, Yoong K, Digger T (2007). Incentive spirometry decreases respiratory complications following major abdominal surgery. Surgeon.

[8] Agostini P, Singh S (2009). Incentive spirometry following thoracic surgery: what should we be doing?. Physiotherapy.

[9] Jaensson M, Dahlberg K, Eriksson M, Nilsson U (2017). Evaluation of postoperative recovery in day surgery patients using a mobile phone application: a multicentre randomized trial. Br J Anaesth.

[10] Hung WW, Ross JS, Farber J, Siu AL (2013). Evaluation of the Mobile Acute Care of the Elderly (MACE) service. JAMA Intern Med.

[11] Semple J, Sharpe S, Murnaghan M, Theodoropoulos J, Metcalfe K (2015). Using a mobile app for monitoring post-operative quality of recovery of patients at home: a feasibility study. JMIR Mhealth Uhealth.

[12] Williams A, Bhatti U, Alam H, Nikolian V (2018). The role of telemedicine in postoperative care. Mhealth.

[13] Dadlani R, Mani S, Mohan D, Rajgopalan N, Thakar S, Aryan S, Hegde AS (2014). The impact of telemedicine in the postoperative care of the neurosurgery patient in an outpatient clinic: a unique perspective of this valuable resource in the developing world--an experience of more than 3000 teleconsultations. World Neurosurg.

[14] Siddle J, Pang P, Weaver C, Weinstein E, O'Donnell D, Arkins T, Miramonti C, CORE team M (2018). Mobile integrated health to reduce post-discharge acute care visits: a pilot study. Am J Emerg Med.

[15] Borsci S, Federici S, Lauriola M (2009). On the dimensionality of the System Usability Scale: a test of alternative measurement models. Cogn Process.

[16] Borsci S, Federici S, Bacci S, Gnaldi M, Bartolucci F (2015). Assessing user satisfaction in the era of user experience: comparison of the SUS, UMUX, and UMUX-LITE as a function of product experience. Int J Hum Comput Stud.

[17] Kortum P, Bangor A (2012). Usability ratings for everyday products measured with the System Usability Scale. Int J Hum Comput Stud.

[18] Sauro J, Lewis JR (2012). Quantifying the User Experience: Practical Statistics for User Research.

[19] Brooke J (1996). SUS-a quick and dirty usability scale. Usability Evaluation in Industry.

[20] Bangor A, Kortum P, Miller J (2009). Journ Usability Stud.

[21] Lehmann M, Monte K, Barach P, Kindler CH (2010). Postoperative patient complaints: a prospective interview study of 12,276 patients. J Clin Anesth.

[22] Faul F, Erdfelder E, Buchner A, Lang AG (2009). Statistical power analyses using G*Power 3.1: tests for correlation and regression analyses. Behav Res Methods.

[23] Overdijkink SB, Velu AV, Rosman AN, van Beukering MD, Kok M, Steegers-Theunissen RP (2018). The usability and effectiveness of mobile health technology-based lifestyle and medical intervention apps supporting health care during pregnancy: systematic review. JMIR Mhealth Uhealth.

[24] Robinson MD, Branham AR, Locklear A, Robertson S, Gridley T (2015). Measuring satisfaction and usability of FaceTime for virtual visits in patients with uncontrolled diabetes. Telemed J E Health.

[25] Ackermann RT, Finch EA, Brizendine E, Zhou H, Marrero DG (2008). Translating the diabetes prevention program into the community. The DEPLOY pilot study. Am J Prev Med.

[26] Diabetes Prevention Program Research Group (2002). The Diabetes Prevention Program (DPP): description of lifestyle intervention. Diabetes Care.

[27] Yu X, Chau JP, Huo L (2018). The effectiveness of traditional Chinese medicine-based lifestyle interventions on biomedical, psychosocial, and behavioral outcomes in individuals with type 2 diabetes: a systematic review with meta-analysis. Int J Nurs Stud.

[28] Xiao L, Yank V, Wilson SR, Lavori PW, Ma J (2013). Two-year weight-loss maintenance in primary care-based Diabetes Prevention Program lifestyle interventions. Nutr Diabetes.

[29] Richards KA, Jones E (2008). Customer relationship management: finding value drivers. Indust Mark Manag.

[30] Soh JY, Cha WC, Chang DK, Hwang JH, Kim K, Rha M, Kwon H (2018). Development and validation of a multidisciplinary mobile care system for patients with advanced gastrointestinal cancer: interventional observation study. JMIR Mhealth Uhealth.

