# Supplementary figures and images for: A Mobile Phone–Based Self-Monitoring Tool for Perioperative Gastric Cancer Patients With Incentive Spirometer: Randomized Controlled Trial
Source: JMIR Mhealth Uhealth. 2019 Feb 19;7(2):e12204. doi: 10.2196/12204 (PMC6399573; doi:10.2196/12204)

Appendix 1. Video clips for incentive spirometer after surgery


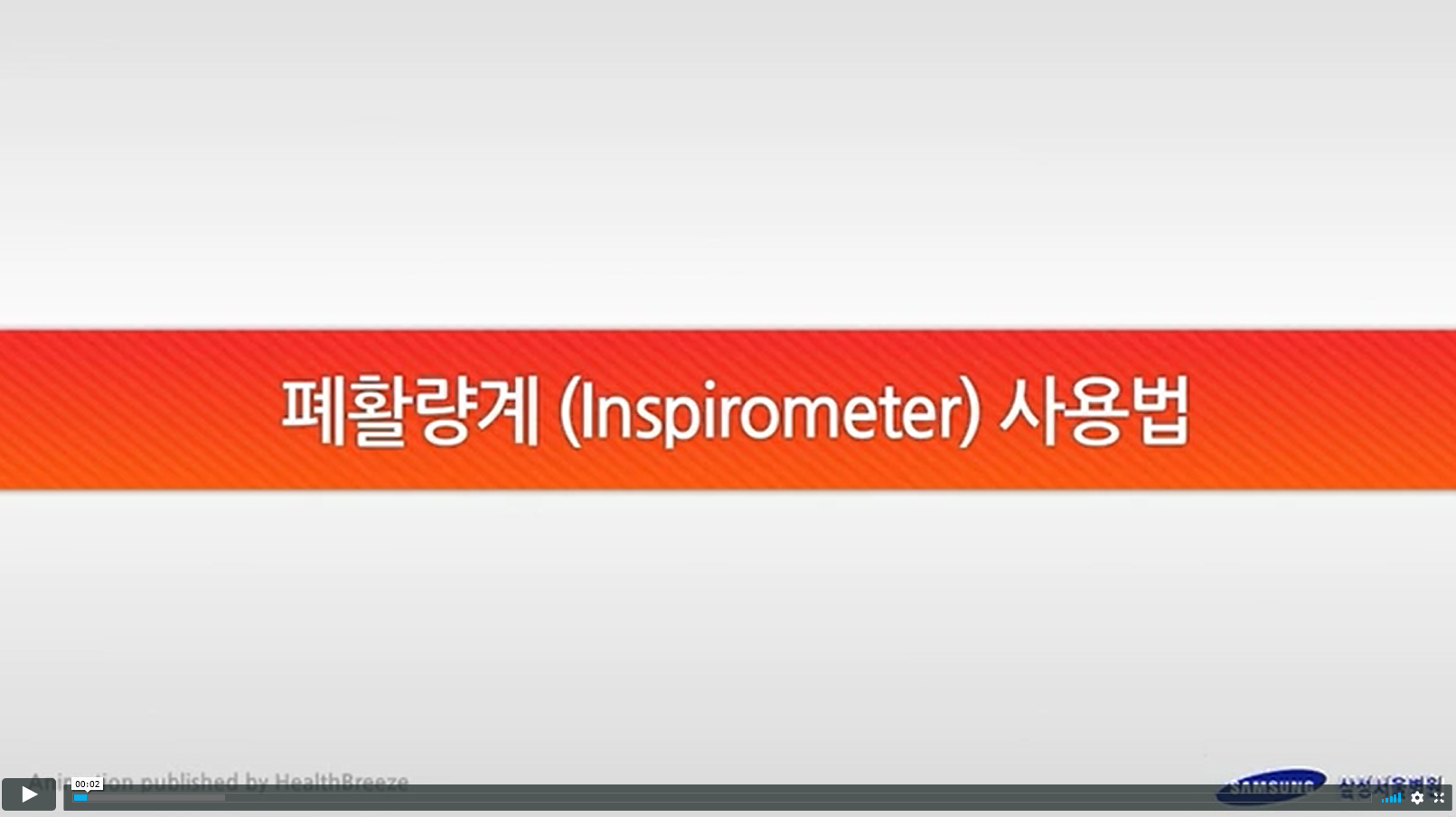


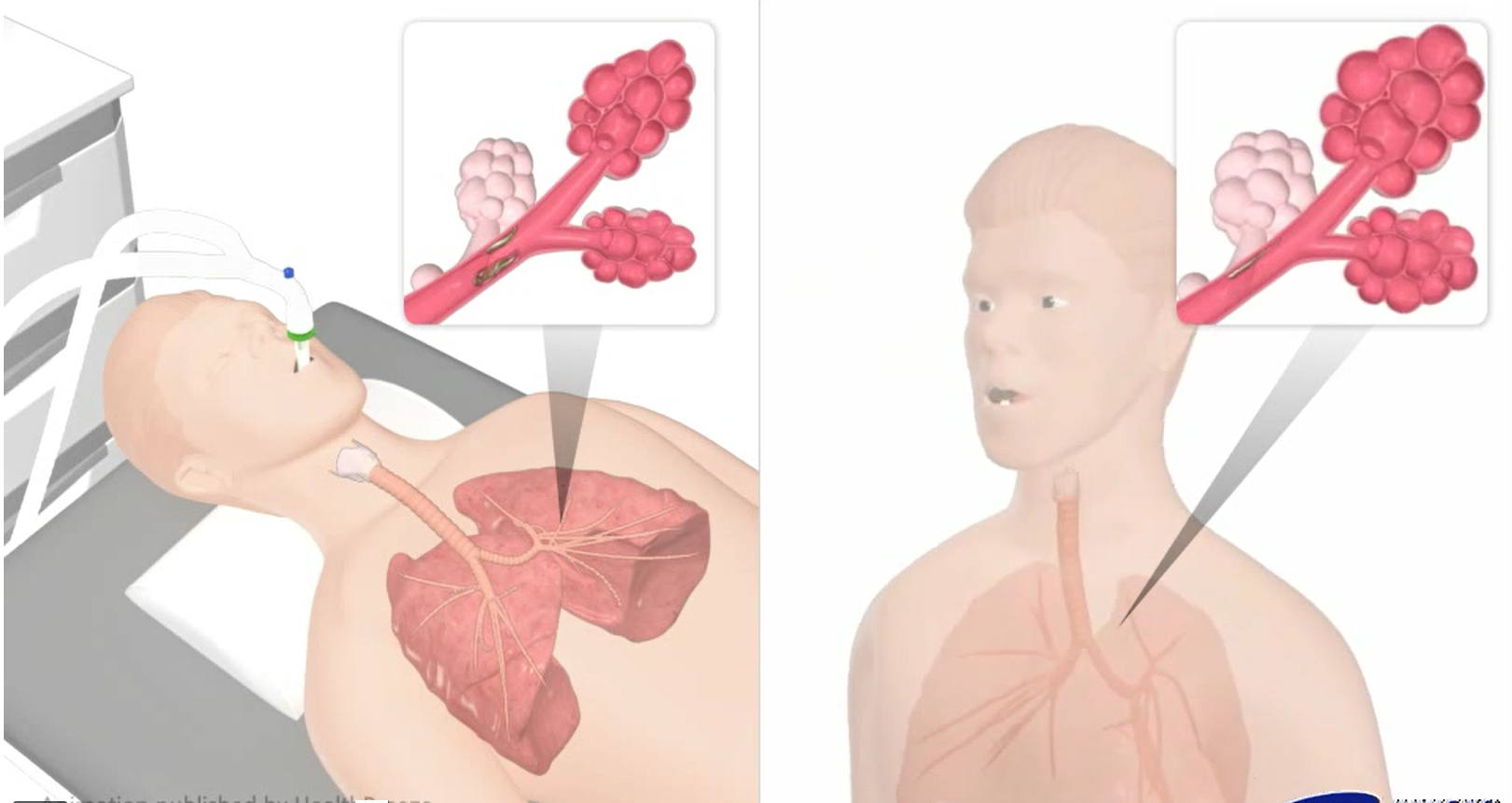


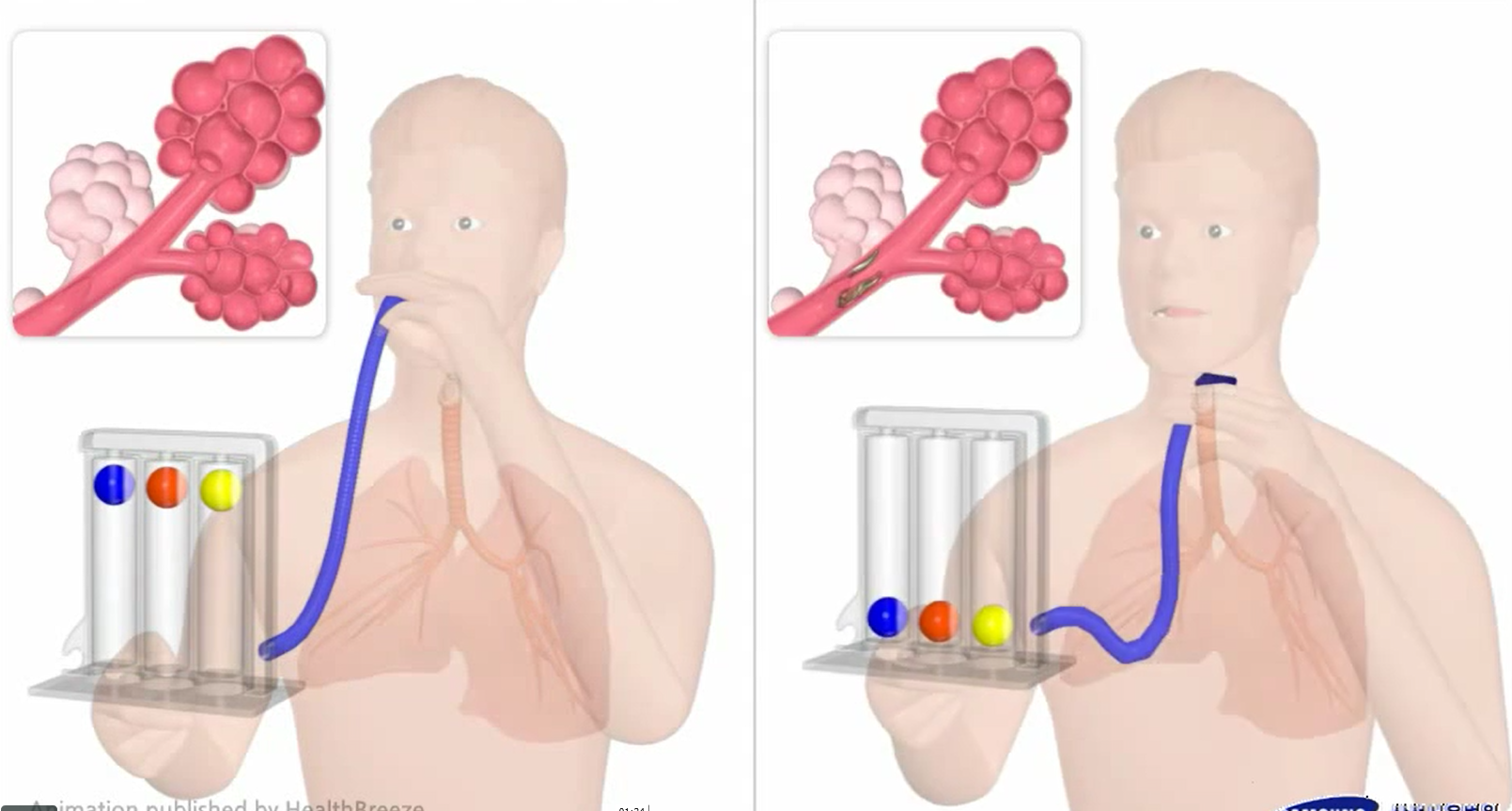

Supplement: Multimedia Appendix 1 [file mhealth_v7i2e12204_app1.docx]
