# Supplementary material for: A Mobile Phone–Based Self-Monitoring Tool for Perioperative Gastric Cancer Patients With Incentive Spirometer: Randomized Controlled Trial
Source: JMIR Mhealth Uhealth. 2019 Feb 19;7(2):e12204. doi: 10.2196/12204 (PMC6399573; doi:10.2196/12204)
Supplement: Multimedia Appendix 2 [file mhealth_v7i2e12204_app2.docx]

Appendix2. Co-morbidities between open surgery or robotic and laparoscopic procedure

|  | Open surgery | Robotic and laparoscopic |
| --- | --- | --- |
|  | N=5 | N=11 |
| Aplastic anemia | 0 | 1 |
| Benign Prostate Hypertrophy | 0 | 1 |
| Diabetes Mellitus | 1 | 3 |
| Dyslipidemia | 0 | 1 |
| Hypertension | 4 | 8 |
| Hypercholesterolemia | 1 | 0 |
| Tuberculosis | 0 | 1 |
